# Frequency–Direction Coupling in the Glass Transition Response of Thermally Aged Wet-Layup Unidirectional Carbon/Epoxy Composites

**DOI:** 10.3390/polym18060680

**Published:** 2026-03-11

**Authors:** Kruthika Kokku, Rabina Acharya, Vistasp M. Karbhari

**Affiliations:** 1 Department of Civil Engineering, University of Texas Arlington, Arlington, TX 76019, USA; 2 Department of Mechanical and Aerospace Engineering, University of Texas Arlington, Arlington, TX 76019, USA

**Keywords:** DMTA, carbon/epoxy, degradation, post-cure, glass transition temperature, thermal aging, frequency dependence, multifrequency, fiber-matrix interphase, wet layup

## Abstract

Dynamic mechanical thermal analysis (DMTA) is widely used to assess the effects of process- and environment-induced changes in polymer matrix composites, with the glass transition temperature (T_g_) often reported from the tan d peak at a single excitation frequency. However, such an approach neglects the inherently kinetic nature of the glass transition and may obscure thermally induced changes in relaxation response. Multi-frequency DMTA was employed to investigate the evolution of glass transition response of a wet-layup unidirectional carbon/epoxy composite subjected to thermal aging at temperatures ranging from 66 °C to 260 °C for periods up to 72 h, using unexposed (23 °C) results as an ambient baseline reference. Tests were conducted using a single cantilever mode in both longitudinal and transverse configurations over a range of excitation frequencies from 0.3 to 30 Hz. Results demonstrate that thermal exposure affects not only the absolute value of the glass transition temperature, but also its frequency sensitivity and directional dependence. A frequency sensitivity parameter and a directional amplification factor are introduced to quantify frequency–direction coupling. While post-cure-dominated aging regimes exhibit relatively stable coupling behavior, degradation-dominated conditions at elevated temperatures and longer periods of thermal exposure lead to pronounced increases in transverse frequency sensitivity, which reflects early evolution of matrix- and interphase-level deterioration. These findings highlight the value of multi-frequency DMTA with tests in both primary directions for the mechanistic assessment of effects of thermo-oxidative response in polymer matrix composites.

## 1. Introduction

Fiber-reinforced polymer (FRP) composites are commonly used in aerospace, marine, and offshore applications due to their lightweight, high specific strength and stiffness characteristics, corrosion resistance, and potentially high service life durability, tailorability, and design flexibility. In addition, in sectors such as civil infrastructure, the ease of placement in the field, especially as related to rehabilitation and retrofit is resulting in its increased use in conjunction with the wet layup process. Many of these applications are governed by requirements of service temperatures and thermal aging, in addition to hygrothermal exposures. In such systems, time-dependent thermomechanical and thermochemical changes in the polymer matrix and the fiber matrix interphase control attributes such as stiffness and strength retention, damage initiation and growth, stress redistribution, and failure, and thereby govern long-term performance and durability.

The glass transition temperature, T_g_, is a central parameter linking the polymer network structure to temperature-dependent behavior. Below the T_g_, the polymer matrix exhibits a glassy response characterized by limited segmental mobility and high relative stiffness, whereas above the T_g_, it transitions to a rubbery and more compliant state associated with reduced modulus, increased damping, and accelerated mechanisms of time-dependent deformation such as stress relaxation. Because matrix-dominated mechanisms control many durability critical responses in composites, especially as related to transverse cracking and intralaminar/interlaminar shear, the glass transition temperature is often used as a practical upper threshold in routine design.

While T_g_ is often considered a threshold for response, and as a material characteristic, it must be noted that it is not an intrinsic or immutable material constant, especially in thermoset polymers and composites subjected to thermal exposure. Elevated temperature exposure can activate multiple competing mechanisms whose relative influence depends on both exposure temperature and duration. At moderate temperatures and periods of exposure, continued cure and physical aging may increase effective crosslink density, reduce free volume, and shift the glass transition temperature to higher temperature levels. Epoxies cured under ambient and moderate temperature conditions show post-cure effects in terms of increases in T_g_ and mechanical properties as a function of exposure temperature [1,2]. At high temperatures of exposure, or longer exposure times, thermos-oxidative degradation becomes the dominant factor, leading to chain scission, formation of oxidation products, embrittlement, microcracking, degradation of the fiber–matrix interphase, and progressive loss of resin-dominated mechanical integrity [3,4,5,6,7]. Extensive studies on polymers and polymer-based composites, especially those processed under ambient or moderate-temperature cure conditions, demonstrate that these competing processes often produce non-monotonic evolution of T_g_, with early increases followed by stabilization, or decline, as degradation progresses [3,8,9].

Experimental investigation of carbon/epoxy and glass/epoxy systems has shown that thermal aging in air can alter stiffness, strength, and fracture behavior, frequently exhibiting early-stage property increases consistent with post-cure, followed by degradation at more aggressive exposure conditions of time and temperature levels. Thermomechanical indicators associated with matrix relaxation, such as T_g_ and damping behavior, provide a mechanistically meaningful bridge between chemical degradation processes and performance-relevant mechanical property changes. In the case of unidirectional composites, the interpretation of T_g_ is complicated by strong material anisotropy and the role of the fiber–matrix interphase. In longitudinal loading configurations where fibers are aligned along the test span, the polymer matrix is strongly constrained by the fibers, whereas in the transverse direction, where fibers are perpendicular to the span, or in shear-dominated configurations, deformation is governed primarily by the matrix and the interphase. Because thermal oxidation and related degradation mechanisms preferentially affect the polymer phase and the interphase [10], exposure-induced changes in T_g_ and viscoelastic response will differ depending on loading direction. Despite the importance of fiber orientation, there is limited data on the effect of orientation and layup on T_g_ and dynamic mechanical properties of composites [11]. Although T_g_ is associated with matrix segmental relaxation, the measured manifestation of T_g_ in terms of aspects such as peak temperatures, peak width, and damping magnitude can differ by direction of reinforcing fibers because constraint and interphase condition-governed matrix response is coupled to composites level response. Damage processes at this level are not simply functions of temperature alone. Rather, they depend strongly on temperature–time history, availability, and diffusion of oxygen into the composite, and subsequent diffusion–reaction coupling, all of which result in gradients and changing constraint states that create complex mechanistic and chemical interactions.

While dynamic mechanical thermal analysis (DMTA) provides a powerful and sensitive way to interrogate macromolecular changes through viscoelastic transitions, it must be remembered that the T_g_ obtained from such analysis is not an intrinsic constant. Rather, it depends on the chosen parameter (onset of storage modulus, peak of loss modulus, and tan δ peak) [12] and on the excitation frequency [13], since T_g_ reflects a kinetic relaxation process. The tan δ peak, or damping factor, is extremely sensitive to molecular mobility and energy dissipation and is often considered to provide the clearest indication of the maximum point of damping during the transition. Since it marks the highest rate of energy loss (viscous mode) to the stored (elastic mode), it is typically the highest of the three common DMTA values, with the one from the E’ onset being the lowest and that from the E” peak being the intermediate. The aspect of frequency dependence is widely recognized in DMTA practice, and unambiguous reporting requires specification of both the T_g_ definition and the oscillation frequency. More fundamentally, the frequency–temperature equivalence underpinning viscoelasticity (e.g., time temperature superposition concepts) implies that shifting the oscillation frequency changes the apparent transition temperature associated with a given relaxation time scale. Multi-frequency DMTA, therefore, provides access not only to T_g_ but to the sensitivity of the transition to frequency, providing information that is directly connected to the breadth and evolution of the relaxation spectrum and can be parameterized in ways that are useful for the differentiation of mechanisms and for model development. Classic DMTA studies have shown that multi-frequency testing enables the extraction of quantities such as effective activation energy associated with glass transition relaxation in epoxies [13]. Despite this, most DMTA studies on the aging of epoxies are conducted using the single-frequency mode of testing [14]. Often, this is because of its relative simplicity, standardization, and alignment to the qualification standards, such as ASTM D7028 [15]. While convenient and standardized, this approach implicitly assumes that thermal exposure only affects the position of the glass transition, not the nature of the relaxation process itself. Under thermal aging, however, oxidation, interphase degradation, and microstructural damage can broaden the relaxation spectrum, change rate sensitivity, and compromise the physical meaning of a single T_g_ value. Two aging conditions may therefore exhibit similar glass transition temperatures at a single frequency while possessing fundamentally different viscoelastic kinetics and damage states. For example, one could be consistent with the post-cure-driven narrowing or shift of the relaxation distribution, while another could be consistent with oxidation-driven heterogeneity, constraint loss, and progressive loss of a well-defined tan δ peak. These distinctions matter for the assessment of durability and structural integrity because they reflect differences in the evolving polymer network and the interphase constraint state, which, in turn, governs the matrix-dominated strength, fracture, and damage tolerance.

Multi-frequency DMTA provides the means to overcome this limitation by enabling direct assessment of how T_g_ shifts with excitation frequency. By examining the frequency sensitivity of T_g_, it is possible to infer changes in the relaxation kinetics, heterogeneity, and mechanical constraint associated with thermal exposure. Prior studies on epoxies and polymer composites have demonstrated the utility of multi-frequency DMTA for extracting activation energies associated with glass transition relaxation and for assessing environmental effects, including thermal and hygrothermal conditioning [16,17]. Environmental exposure has been shown to modify not only the absolute T_g_, but also the frequency dependence, breadth, and integrity of the tan d peak, all features that reflect evolving network structure and damage development. While dynamic mechanical analysis is widely used to study thermally aged polymer composites, most published studies rely on single-frequency measurements and limited laminate orientations [11], and its systematic use across multiple excitation frequencies and orientations for composites exposed to elevated temperature regimes remains limited. In particular, the combined influence of exposure temperature, exposure time, excitation frequency, and loading direction on T_g_ evolution has not been comprehensively examined using quantitative coupling metrics.

The objective of the present work is therefore to establish and apply a frequency–direction coupling framework for interpreting T_g_ evolution in a unidirectional carbon/epoxy composite subjected to elevated temperature exposure across a range of temperature–time conditions. The composite is characterized by multi-frequency DMTA in single cantilever mode in both longitudinal and transverse configurations over excitation frequencies of 0.3 to 30 Hz. Building on prior work that demonstrated that multi-frequency DMA can sensitively capture aging-induced changes in composite response [16], quantitative metrics are introduced to separate absolute T_g_ shifts from changes in frequency sensitivity and directional amplification.

The work considers a deliberately wide experimental matrix spanning temperatures, exposure times, excitation frequencies, and material directionality. Given the resulting number of conditions, a single specimen was evaluated at each temperature–time state. The objective was to capture systematic trends in the evolution of the glass transition temperature across a broad exposure matrix in both longitudinal and transverse directions of the unidirectional composite. Baseline measurements indicated a T_g_ repeatability with the ±2–3° range consistent with typical DMTA repeatability in polymers and polymer composites. Rather than assessing statistical replicates, the analysis focuses on the evaluation of internally consistent and physically interpretable metrics of frequency sensitivity parameters and a dynamic amplification factor with peak-anchored and cross-sectional state comparisons to extract trends and interpretations of the material’s response. While previous studies have investigated the effect of thermal exposure on residual mechanical properties and glass transition temperature, the interaction between frequency-dependent viscoelastic response and material anisotropy has received limited attention. This work introduces two quantitative descriptors to characterize how frequency-dependent T_g_ response evolves for the longitudinal and transverse direction specimens of a unidirectional carbon/epoxy composite, providing a framework for assessing post-cure and deterioration effects as well as the competition between the associated sets of mechanisms.

## 2. Materials and Experimental Methods

Two layers of a unidirectional carbon fabric with 12k untwisted aligned tows of 644 g/m^2^ aerial weight were impregnated using the wet-layup process with a difunctional Bisphenol A-epichlorohydrin-derived liquid epoxy (Epon 828-based) using a polyetheramine-modified polyoxypropylenediamine (Jeffamine D-series). Following procedures used in the field for rehabilitation of civil infrastructure systems/components, only manual roller-based pressure was used, and cure was achieved through exposure to ambient temperature for seven days at 30% RH. In order to ensure a uniform baseline of cure progression for the current investigation, all fabricated panels were conditioned for 72 h at 60 °C. The final laminate thickness was approximately 3 mm. Fiber volume fraction was found to be 49.8% following acid digestion procedures per ASTM D3171 [18]. Void content was noted to vary between 2 and 3%.

Specimens of nominal dimensions of 8.8 mm width and 34 mm in length were cut in both the longitudinal (i.e., fibers along the longer dimension) and transverse (i.e., fibers along the smaller dimension) directions and then exposed to temperatures between 150 °F and 500 °F at intervals of 50 °F, i.e., at 66 °C, 93 °C, 121 °C, 149 °C, 177 °C, 204 °C, 232 °C, and 260 °C, for periods of time ranging from one hour to 72 h. Specimens were placed in furnaces with sufficient spacing to permit unrestricted air access to all exposed surfaces, thereby allowing oxygen diffusion during exposure. Temperature uniformity within the furnace was verified prior to placement of specimens at the specified temperature for the required test period. At the nominal thickness of 3 mm, oxidation during thermal exposure is expected to be surface-dominated at low–intermediate temperatures at lower periods of time, and increasingly through thickness at higher temperatures and overall longer periods of exposure. Specimens were removed after the desired period and allowed to cool down prior to being tested in cantilever mode in a Rheometric Scientific Dynamic Mechanical Thermal Analyzer in multi-frequency mode at 0.3, 1, 3, 10, and 30 Hz at a heating rate of 2 °C/minute and an imposed strain of 0.01%. All frequency sweeps were conducted within the linear elastic regime, verified through preliminary strain-amplitude checks, ensuring that frequency-dependent trends reflect material relaxation behavior rather than non-linear response. Exposure was limited to 24 h at 260 °C in the longitudinal direction and 16 h at the same temperature in the transverse direction due to severe degradation beyond these levels, leading to significant mass loss and geometric instability beyond these points. It should be noted that mass loss as a result of thermal exposure increases with both temperature and time of exposure, with changes between adjacent intervals being incremental and small. However, the loss after periods of 24, 48, and 72 h at 260 °C was 11%, 15.6%, and 18.2%, respectively, all substantially greater than losses at other temperature–time combinations, indicating severe degradation of the epoxy network, including through mechanisms such as chain scission, oxidation, and volatilization of degradation products. As elucidated later in Section 3, there are already dramatic drops in T_g_ at this temperature, even at lower periods of exposure. Previous studies on ±45 off-axis tension characterization have reported the inability to conduct valid tests at these conditions due to extensive twisting resulting from distortion and interlayer delamination [19]. Because thermal exposures were completed prior to DMTA testing and all specimens were tested using the same heating rate, the shift in T_g_ can be directly attributable to prior structural evolution rather than to changes from the test itself. The effects of frequency are also intrinsic viscoelastic effects rather than being artifacts of the test procedure. For the purposes of consistency, the glass transition temperature is defined as the peak of the tan d curve for each frequency and configuration. The investigation of systematic trends across frequency, direction, temperature, and time period provides a large set of data for the period for the development of a comprehensive mechanistic insight of the operative phenomena and effects. the operative phenomena and effects. Data for the same set of excitation frequencies and test directions obtained under unexposed conditions (23 °C) are used as a reference control.

## 3. Results and Discussion

### 3.1. T_g_ Evolution at 1 Hz

**Figure 1:**
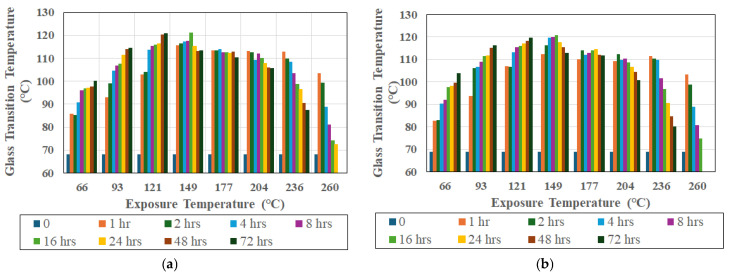
Evolution of glass transition temperature at 1 Hz as a function of aging time and exposure temperature. (**a**) Longitudinal direction. (**b**) Transverse direction.

It should be emphasized that ambient cure systems retain a degree of residual cure potential after fabrication, due to which, when exposed to elevated temperatures, additional cross-linking occurs, resulting in a rapid increase in T_g_. When exposed to similar temperature regimes, the T_g_ of a fully cured system would remain relatively stable until the initiation of deteriorative mechanisms. As shown in Figure 1a,b, at the reference frequency of 1 Hz, T_g_ increases rapidly at early aging times for specimens tested in the longitudinal and transverse directions.

The increase continues for temperatures up to 121 °C, which is consistent with both post-cure and network densification, plateauing at higher times of exposure. The increase in T_g_ due to post-cure was confirmed in an earlier study [19] through the use of differential scanning calorimetry, which showed similar trends. At intermediate temperatures of 149 °C and 177 °C, the initial increase is followed by a minor decrease, and at temperatures greater than or equal to 204 °C, the initial sharp increase is followed by a clear decline with increasing exposure time, indicating the onset of degradation-dominated behavior. Directional differences in T_g_ were observed at all temperatures, with the magnitude of the transverse and longitudinal T_g_ offset evolving with exposure temperature and time, underscoring the anisotropic nature of the viscoelastic response. The maximum increase across all temperatures is seen after 1 h of aging. At temperatures between 66 °C and 121 °C, there is no decline, even at the longest period of exposure of 72 h. The highest levels of T_g_ of 121.17 °C in the longitudinal direction and 120.8 °C in the transverse direction are recorded as a result of exposure at 149 °C, after an exposure period of 16 h, representing increases of 52.95 °C and 51.9 °C over the baseline for longitudinal and transverse directions, respectively. Tsotsis reported similar increases of roughly 50 °C from the unaged value of the glass transition temperature of both epoxy resins and carbon/epoxy composites [20]. It is of interest to note that at the recorded rate of increase in T_g_ of 0.3 °C/hour in the final period of aging at 121 °C it would take between 701.7 and 225.3 h to reach the same peak T_g_ level if exposures at 66 °C and 93 °C, respectively, were continued, emphasizing the slow rate of increase at the longer periods of time at lower temperatures where a near-asymptotic response is seen. While not investigated further in this paper, this trend does suggest that fairly long service lives could be expected at operational exposure levels below the peak T_g_ recorded, i.e., up to about 121 °C.

### 3.2. Frequency Dependence of T_g_ and Frequency Sensitivity Parameter

**Figure 2:**
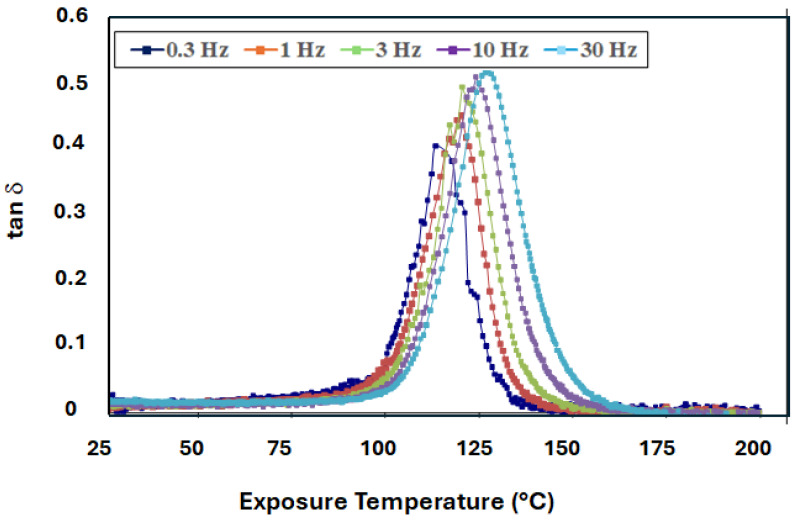
Effect of frequency of excitation on loss modulus curve at T_g_ (exposed to 121 °C for 48 h).

For all temperatures of exposure considered, in both loading configurations, T_g_ determined from the tan d peak is seen to increase with frequency of excitation, which is consistent with the kinetic nature of the glass transition in polymer networks and polymer composites [16,17,21,22]. An example of the trend, as a result of testing in the longitudinal direction after exposure to 121 °C for 48 h, is shown in Figure 2. As can be seen, the glass transition temperature increases from 115.5 °C, as measured at a frequency of 0.3 Hz, to 127.4 °C at 30 Hz. In this case, both the T_g_ and height of the tan d peak increase.

**Figure 3:**
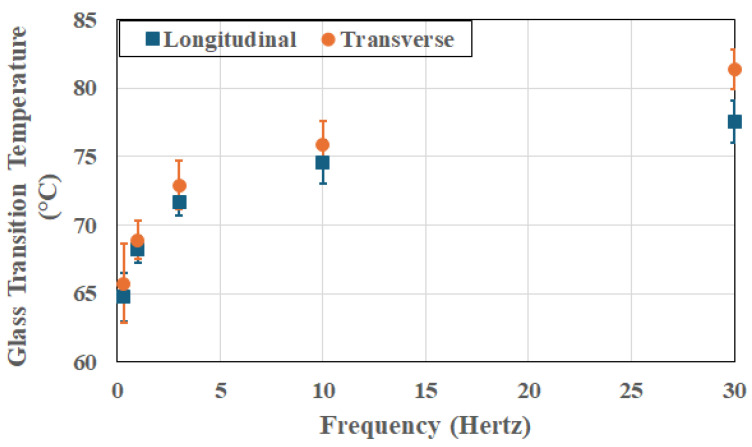
Change in T_g_ recorded at ambient temperatures (i.e., without thermal exposure) as a function of frequency of excitation and direction of testing.

**Figure 4:**
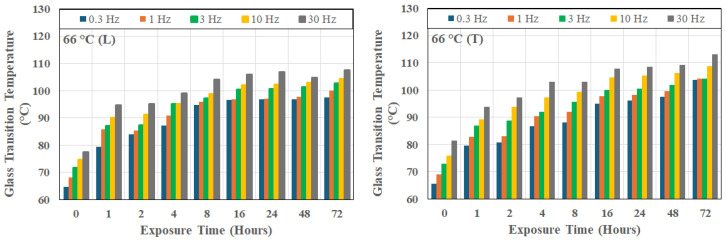
Change in glass transition temperature as a function of time of exposure at 66 °C and frequency of excitation; L: longitudinal direction, and T: transverse direction.

**Figure 5:**
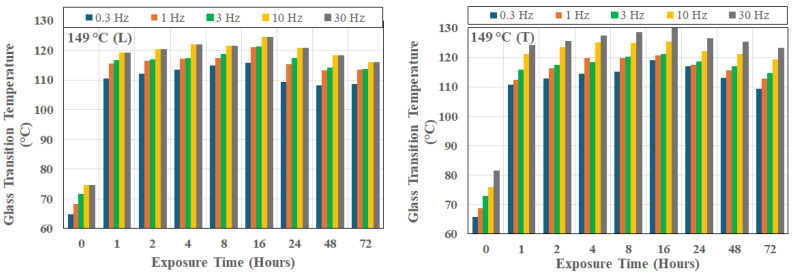
Change in glass transition temperature as a function of time of exposure at 149 °C and frequency of excitation; L: longitudinal direction, and T: transverse direction.

**Figure 6:**
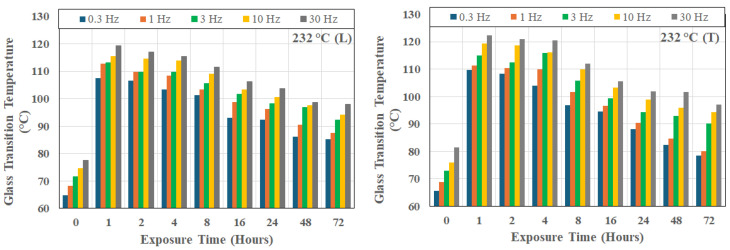
Change in glass transition temperature as a function of time of exposure at 232 °C and frequency of excitation; L: longitudinal direction, and T: transverse direction.

It is of interest to note that this trend is seen even in the unexposed state, as shown in Figure 3, which also shows that the unidirectional composite has intrinsic anisotropic relaxation kinetics even prior to thermal aging, with the transverse setup leading to consistently higher glass transition temperatures, as would be expected due to the decreased fiber constraint. The scatter bounds relate to the change across the 72 h period. Figure 4, Figure 5 and Figure 6 show the frequency dependence of T_g_s at three representative temperatures of 66 °C, 149 °C, and 232 °C, respectively. As can be seen, at a given exposure temperature and time, T_g_ values measured in the transverse direction (as shown by the right-hand-side graph in each Figure) were generally higher than those measured in the longitudinal direction at the same frequency, with the difference increasing systematically with the excitation frequency.

At lower levels of thermal exposure (T ≤ 121 °C), T_g_ increases gradually with time of exposure across all frequencies, reaching a maximum asymptotically at the longest duration of exposure of the investigation, i.e., 72 h. It is of interest to note that the value of peak T_g_ increases with temperature in this range of exposure temperatures with values of 100.12 °C, 114.41 °C, and 120.85 °C at 66 °C, 93 °C and 121 °C, respectively, as measured from longitudinal tests at 1 Hz.

In the moderate temperature range between 149 °C (Figure 5) and 232 °C (Figure 6), the time required to attain the maximum T_g_ decreases rapidly with peaks attained at progressively shorter times as temperature of exposure increases. At, and beyond, 232 °C, as shown by Figure 5, T_g_ decreases rapidly after very early attainment of a peak.

To quantify this behavior, a frequency sensitivity parameter (FSP) was defined as the slope of T_g_ versus log_10_ (f), such that:

(1) $$FSP\left(t,T,d\right)={\left.\frac{dT_{g}}{dlog10(f)}\right\rceil }_{t,T,d}$$

over the range of 0.3 to 30 Hz, where f is the frequency, T is the temperature of exposure, t is the time of exposure, and d is the direction of the specimen (either longitudinal (L) or transverse (T)). Across the frequency range investigated (0.3–30 Hz), the glass transition temperature exhibits an approximately linear dependence on log_10_ (f), enabling the use of a single slope parameter as a descriptor. This parameter provides a local kinetic description of the glass transition, enabling direct comparison across aging conditions and between the longitudinal and transverse configurations of testing. Given the scope of the experimental matrix, a single specimen was tested for each temperature–time condition. Consequently, frequency- and direction-dependent responses discussed in the next sections must be treated as internally consistent descriptors of material state. A criterion for assessing uncertainty is introduced, and the uncertainty bands associated with frequency sensitivity parameters, therefore, reflect confidence levels in that vein and should not be viewed as specimen level variability. Given the difference in evolution of the effects of heat based on the constraint from the directionality of the fibers, it is of interest to assess this from a directional amplification factor (DAF), defined as:

(2) $$DAF=\frac{{FSP}_{T}}{{FSP}_{L}}$$

where the subscripts T and L refer to the direction of the fibers related to the test span. Values greater than unity indicate enhanced frequency sensitivity in the transverse configuration and emphasize the increase in strong frequency–directional coupling as the level of degradation increases due to the increased relaxation heterogeneity and network deterioration in the matrix-dominated configuration. Because DAF is a metric based on a ratio, it is most meaningful when there are distinct differences between the longitudinal and transverse frequency sensitivities. Values approaching unity at low sensitivities should therefore be interpreted qualitatively rather than strictly quantitatively. The combination of FSP values in the two directions of testing, and the DAF, can provide significant information related to the evolution of T_g_ and the effects of frequency and fiber constraint as a function of exposure temperature, with FSP reflecting the intrinsic kinetic sensitivity of the densified network and the DAF indicating how constraint differs by direction at the same network state. It is emphasized that FSP and DAF parameters are intended primarily as phenomenological descriptors that enable comparison across exposure conditions and material directions. While these parameters reflect underlying viscoelastic relaxation mechanisms, they are not intended to serve as intrinsic material constants.

As noted previously [8], thermal exposure results in a competition between the phenomena of thermally induced progression of cure (post-cure) and deterioration, with the overall behavior generally being in three zones representing: (a) domination of post-cure-related mechanisms prior to attainment of a peak T_g_ under the specific conditions of temperature and time of exposure; (b) the domination of mechanisms of deterioration after the attainment of peak T_g_; and (c) the transition region where there is competition between these extremes. In line with this, it is worth further assessing details of response at the peak T_g_ attained across all conditions of thermal exposure. Table 1 lists the time and peak temperature pertaining to T_g_ at each temperature of exposure for tests conducted in longitudinal and transverse directions. It should be noted that while the peak in the longitudinal and transverse directions is attained at the same time, irrespective of frequency of excitation in most cases, there is some variation within sets. In this case, results pertaining to temperature at both time periods are shown for completeness. The time taken to attain the peak T_g_ decreases with an increase in the temperature of exposure. At temperatures of 66 °C, 93 °C, and 121 °C, values attained at the 72 level of exposure represent the maximum reached within the period of exposure, with trends indicating the potential of continued slow increase at longer periods, which were not investigated in the current study. Because the time required to reach peak T_g_ varies strongly with exposure temperature, frequency sensitivity and directional amplification are first considered at the aging condition corresponding to peak T_g_ at each temperature of exposure, which facilitates comparison of the material at similar stages of exposure effect across temperature, providing a physically meaningful baseline for assessing frequency–direction coupling.

**Figure 7:**
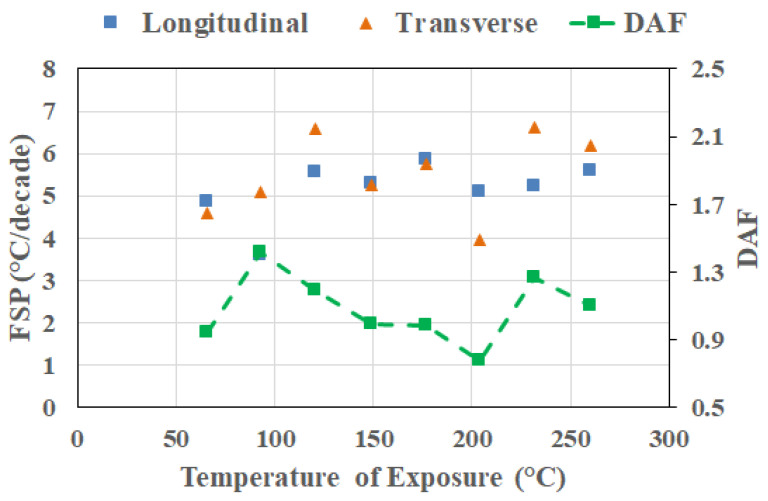
Frequency sensitivity parameter (FSP) and direction amplification factor evaluated at the exposure condition related to attainment of peak T_g_.

Figure 7 presents the frequency sensitivity parameters and the direction amplification factor evaluated at the exposure condition corresponding to the peak T_g_ attained at each exposure temperature and direction. By anchoring the analysis at the peak T_g_, the comparison aligns the material states across temperatures, eliminating potential ambiguity associated with fixed-time (i.e., cross-section) comparisons that consider pre-peak, near-peak/peak, and post-peak conditions together. This also provides a physically aligned reference state that enables a more meaningful interpretation of subsequent cross-sectional responses. It is emphasized that the peak T_g_ represents a kinetic milestone in the thermal aging process corresponding to the saturation of post-cure-driven network densification prior to the dominance of mechanisms of degradation.

As seen in Figure 7, both longitudinal FSP (FSP_L_) and transverse FSP (FSP_T_) values exhibit non-monotonic variation with exposure temperature. Longitudinal FSP presents a tighter band with modest variation with temperature, indicating that once post-cure reaches an asymptotic saturation level, the frequency dependence of T_g_ along the fiber direction is strongly constrained and only weakly sensitive to the severity of exposure. In most cases, FSP_L_ values are similar to, or lower than, FSP_T_ values. With the exception of 121 °C and 204 °C, for reasons that will be discussed later, FSP values show an overall increasing trend with exposure temperature, indicating that, in general, even before initiation of extensive degradation, the transverse relaxation process increases in sensitivity to excitation frequency with increases in the temperature of exposure, reflecting the greater role of resin-dominated deformation and interphase-controlled relaxation in the transverse direction.

Similarly, the peak-anchored DAF values do not vary monotonically with exposure temperature, fluctuating above and below unity across the investigated range, showing an increase till 99 °C followed by a decrease till 204 °C, and a subsequent increase, which corresponds to the change in transition zones for interlaminar and intralaminar shear reported earlier by Acharya and Karbhari [8]. This observation emphasizes that directional amplification at the peak T_g_ condition is not governed solely by exposure temperature, but also by the relative balance of kinetic processes active at the moment the peak is reached in each direction. In particular, differences in the timing of attainment of peak T_g_ between longitudinal and transverse directions at some temperatures of exposure indicate that the peak condition can correspond to slightly different balances of network densification and incipient degradation in the two directions. Importantly, it should be noted that the absence of monotonicity in the DAF does not diminish the value of the peak-anchored analysis. Rather, it demonstrates that even when compared at an equivalent T_g_ milestone, frequency–direction coupling remains sensitive to the history-dependent evolution of constraint and relaxation heterogeneity. This reinforces the central premise of the study: frequency sensitivity and directional amplification capture aspects of thermally induced evolution that are not encoded in T_g_ values alone.

From a broader perspective, the peak-anchored FSP and the DAF results suggest that exposure temperature controls the kinetic pathway by which the polymer network reaches its densified state, with important consequences for subsequent degradation behavior. The enhanced transverse frequency sensitivity observed at high-temperature peak conditions likely predisposes the material to more pronounced rate-dependent degradation and mechanical property loss at later stages. As such, peak-anchored frequency–direction metrics offer a physically grounded means of assessing the severity of thermal exposure beyond conventional T_g_-based descriptors.

It is important that the post-peak evolution of frequency–direction coupling is interpreted relative to the exposure temperature-dependent attainment of peak T_g_ rather than with respect to a common absolute period of exposure. Because T_g_ peak decreases sharply with increasing exposure temperature, from ≥72 h at T ≤ 121 °C to 1–2 h at T ≥ 204 °C, the post-peak regime must be understood to span different periods of time. At lower exposure temperatures the data set primarily captures pre-peak and near-peak response and clearly indicates slow kinetics characterizing both a low ratio of continued polymerization through post-cure and the lack of any substantive deteriorative mechanisms even at extended periods of time, suggesting that within the lowest temperature range thermal exposure causes an increase in T_g_ and the potential effects of deteriorative mechanisms are far less than the positive effects of post-cure, even after an extended period of time. In contrast, at intermediate and high temperatures of exposure, frequency sensitivity evolves systematically beyond the peak T_g_ level in a directionally dependent manner. In these cases, the peak T_g_ level indicates the transition from a post-cure-dominated regime to one dominated by the deteriorative mechanisms of thermal exposure. FSP_L_ values vary moderately with time, indicating that as network densification approaches saturation, further thermal exposure does not substantially change the rate sensitivity of fiber-dominated response, which is in line with the constraint expected by fiber alignment. In contrast, FSP_T_ levels increase or remain elevated with continued exposure at the same temperature.

**Figure 8:**
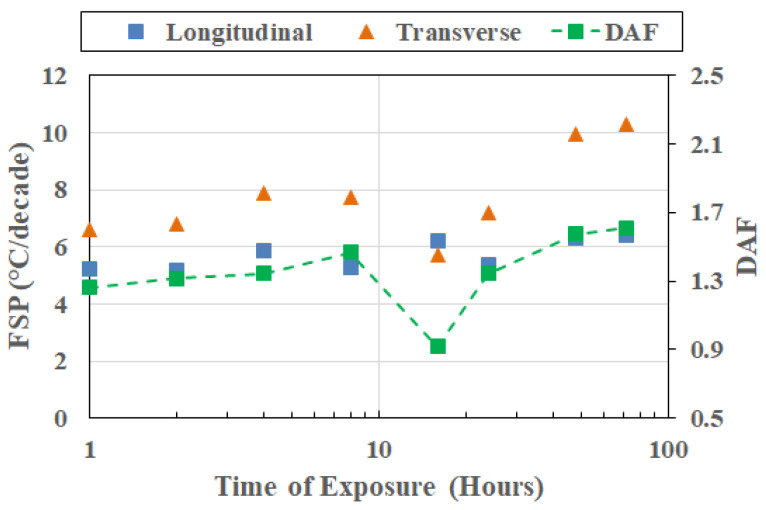
FSP and DAF response at 232 °C.

This divergence in response (as shown in Figure 8 at 232 °C, as an example) reflects the increasing influence of degradation-induced heterogeneity in resin- and interphase-dominated deformation modes by oxidative processes, chain scission, and interphase deterioration, broadening the relaxation spectrum in the transverse, or resin-dominated, direction. Directional amplification thus arises primarily from post-peak evolution in transverse response. Importantly, this amplification is not uniform across temperatures, reinforcing the aspect of frequency–direction coupling being strengthened with progression beyond peak T_g_ rather than being fully established at the peak itself. Given the growth in coupling to transverse degradation phenomena after peak T_g_ attainment, it is of value to assess cross-sectional response (i.e., changes in FSP and DAF as a function of temperature at the same point in time) over the range of exposure temperatures.

Recall that FSP is estimated by linear regression of T_g_ against log_10_(frequency) for each exposure condition and loading direction using data collected at the first excitation frequency in the following form:

(3) $$T_{g}=a+b\;{log}_{10}(f)$$

where b is the slope corresponding to the FSP. It is important, then, to assess the uncertainty associated with this slope since it intrinsically results from a single set of points per condition. The standard error of slope, SE, can be determined following least squares regression as:

(4) $$SE=\sqrt{\frac{1}{n-2}\;\frac{\sum_{i=1}^{n}{\left(T_{g,i}-\hat{T_{g,i}}\right)}^{2}}{\sum_{i=1}^{n}{\left({log}_{10}f_{i}-\bar{{log}_{10}f}\right)}^{2}}}$$

where n is the number of frequencies (5 here), *T_g,i_* are the measured glass transition temperatures, $\hat{Tg,i}$ are the values predicted by the fitted regression line, and $\bar{{log}_{10}f}$ is the mean of the logarithmic frequency values. Equation (4) shows that SE depends on both the scatter of data about the regression line and the span of the frequency domain used. Thus, SE quantifies how precisely the frequency dependence of T_g_ is resolved for an exposure condition, enabling comparison across adjacent conditions to assess whether the changes are statistically meaningful. To do this, the combined standard error of the differences in slope can be determined as:

(5) $${SE}_{C}=\sqrt{{SE}_{FSP,1}^{2}+{SE}_{FSP,2}^{2}}$$

where *SE_FSP_*_,1_ and *SE_FSP_*_,2_ are the standard errors of the two FSP values being compared. We can also define a resolution metric t* such that:

(6) $$t^{*}=\frac{\left|{FSP}_{1}-{FSP}_{2}\right|}{{SE}_{c}}=\frac{∆FSP}{{SE}_{c}}$$

is used as the detectability criterion. Thresholds of t* = 2 and t* = 3 can be adopted as resolution criteria reflecting conventional signal-to-uncertainty benchmarks analogous to 95% and 99% separation levels in classical test statistics, respectively, while avoiding claims of formal statistical significance, such that three bands can be defined as in Table 2.

It should be noted that the value of 2 is taken to be representative of the minimum defensible threshold, whereas that of 3 is an intentionally conservative threshold analogous to the 3s type thresholds (s ≡ standard deviation) commonly used to indicate robust separation from uncertainty, even allowing for a limited number of frequency points, 5, and kinetic complexity. It should be emphasized that SE does not represent specimen-to-specimen variability, but rather the regression uncertainty for that specimen. Hence, SE plays a role analogous to a standard deviation, but for an estimated parameter rather than for raw data, with error bars being analogous to the ±standard error of the FSP. Therefore, mathematically, SE_FSP_ is proportional to the residual standard deviation of the fitted data and inversely proportional to the spread of the frequency values used in the regression. Since it quantifies uncertainty in a kinetic parameter extracted from a single specimen through regression across frequencies, it should be considered as a measure of parameter resolution (reflecting material state) rather than of material variability. As cross-sectional analysis is considered in the discussion ahead, it is important to remember that these comparisons of FSP values across different temperatures at fixed times of exposure inherently mix conditions corresponding to various stages of network evolution, i.e., pre-peak, near-peak, and post-peak periods. In these conditions, apparent differences in FSP can arise either from actual changes in frequency sensitivity or from limitations in the resolution of the slope estimate itself. The incorporation of SE_FSP_ can thus be considered analogous to standard deviation and will be used as such in subsequent Figures, which represent points in the exposure history, to distinguish meaningful trends from the scatter that falls within regression uncertainty. Accounting for SE_FSP_, therefore, prevents over-interpretation of slight differences in FSP and provides a transparent basis for assessing complex cross-sectional trends. More broadly, it ensures that conclusions regarding the evolution of frequency sensitivity and directional amplification are grounded in parameter resolution rather than visual inspection of nominal values alone.

**Figure 9:**
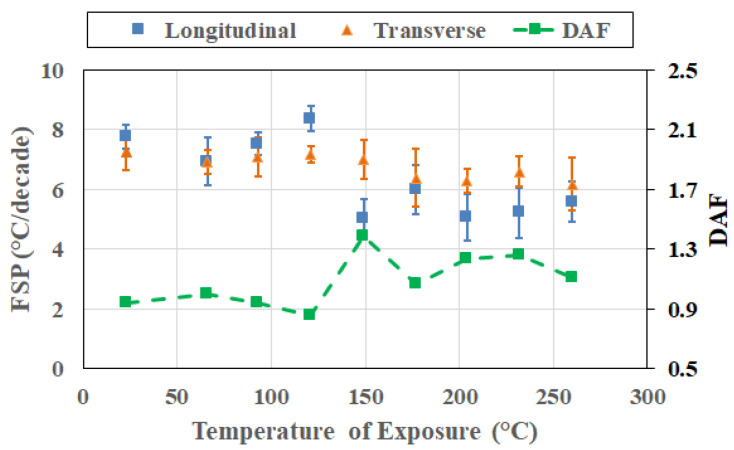
Cross-sectional response after 1 h of thermal exposure as a function of temperature of exposure.

As seen in Figure 9, at the level of 1 h of exposure, longitudinal FSP values are relatively high at lower and intermediate temperatures (e.g., ~7.76 at 23 °C and ~8.37 at 121 °C), reflecting a strong dependence of T_g_ on excitation frequency under conditions dominated by intrinsic segmental kinetics and rapid increases in post-cure levels at these temperatures, which are still below peak T_g_ of 121.17 °C for the overall investigation. At these temperatures, the polymer network remains relatively homogeneous, and frequency sensitivity primarily reflects the thermally activated mobility of fiber-constrained amorphous resin segments. At 149 °C, there is a pronounced drop in longitudinal FSP (~5.04), indicating that elevated temperatures can rapidly alter the relaxation spectrum, reducing the slope of T_g_ versus log_10_(f) through an acceleration of network reorganization at lower temperatures and rapid saturation of post-cure processes. As can be seen from the Figure, and as confirmed through the resolution metric t* in Table 2, the differences are not resolvable in the 23 °C to 121 °C and 149 °C to 260 °C intervals but are marginally resolvable across the full range from 23 °C to 260 °C at t* = 2.77. In comparison, transverse FSP values remain within a fairly narrow band across the entire temperature range, with t* indicating that FSP_T_ comparisons are not resolvable, i.e., the differences are comparable to, or smaller than, the uncertainty in slope estimation. As can be seen from Figure 9, clear directional inversion occurs at 149 °C, where longitudinal FSP exceeds transverse values at low temperatures and then transverse FSP becomes larger at, and after, 149 °C. This suggests that early transverse relaxation is less sensitive to rapid kinetic stiffening but becomes increasingly influenced by temperature-induced changes in constraint effectiveness at higher exposure temperatures. It is noteworthy that the peak DAF value of 1.39 is at 149 °C, which is in congruence with these changes, with the average DAF between 23 °C and 121 °C being 0.959, with a standard deviation of 0.057, whereas that between 177 °C and 260 °C is significantly higher at 1.167 with a standard deviation of 0.098 (but still below the peak DAF value), emphasizing the two different regimes based on time to reach T_g_, as shown in Table 1. While a clear differentiation can be made between the effects of lower temperatures of exposure when post-cure, which, while accelerated, are still increasing after 1 h of exposure, and higher temperatures, where the regime is already transitioning into post peak T deterioration, the 2 h level suggests a greater level of competition between post-cure and deterioration mechanisms, resulting in emerging heterogeneity.

**Figure 10:**
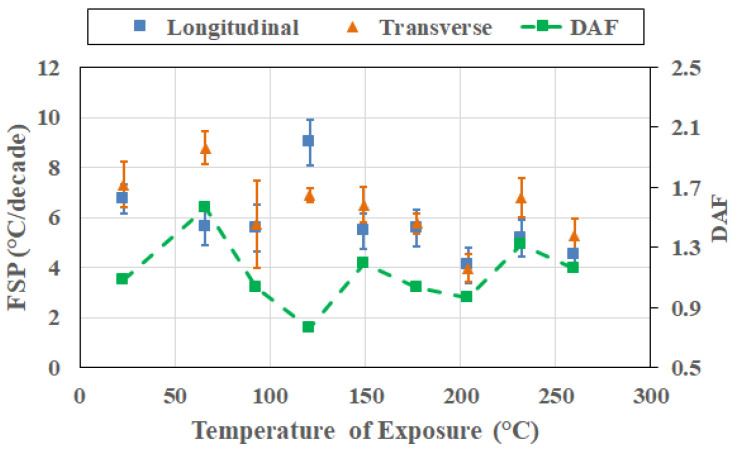
Cross-sectional response after 2 h of thermal exposure as a function of temperature of exposure.

As seen in Figure 10, FSP in both directions demonstrates strong non-monotonic temperature dependence. Longitudinal FSP shows a local maximum at 121 °C (~8.995) followed by a significant drop to levels between 4.09 and 5.6 from 149 °C onwards, which is consistent with enhanced frequency sensitivity during active post-cure progression prior to network saturation. The differences in the ranges between 23 °C and 121 °C and 121 °C and 260 °C are clearly resolvable with t* values of 7.64 and 5.02, respectively. Transverse FSP shows stronger variability, including elevated values at low temperatures (e.g., ~8.79 at 66 °C) and pronounced suppression at higher temperatures (e.g., ~3.97 at 204 °C). On average, the transverse FSP is higher than the longitudinal, with resolvability being clear only between 121 °C and 260 C with t* = 3.82. The direction amplification factor alternates, largely around unity, across the temperature range, which reflects the competition between post-cure, temperature accelerated relaxation, and the onset of microstructural heterogeneity, whereby frequency sensitivity can increase or decrease in ranges depending on how the relaxation spectrum shifts relative to the tan d peak, resulting in a highly condition-dependent directional behavior.

**Figure 11:**
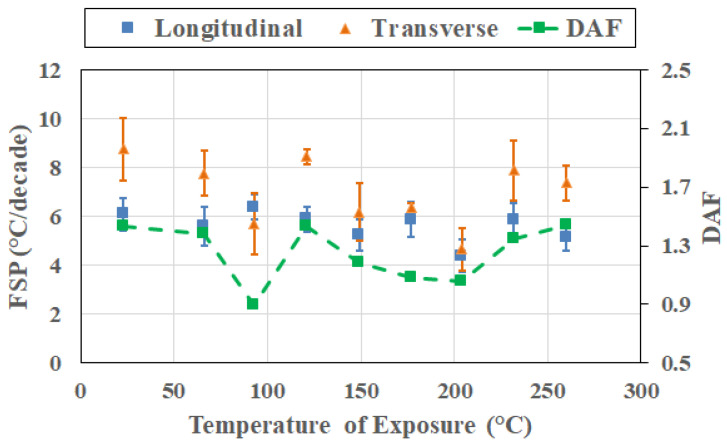
Cross-sectional response after 4 h of thermal exposure as a function of temperature of exposure.

Results from the 4 h exposure levels, as shown in Figure 11, cover a regime that indicates the onset of clearer temperature-dependent directional coupling, i.e., directional asymmetry. The longitudinal FSP remains within moderate levels and shows limited systematic dependence on exposure temperature, indicating that frequency sensitivity along the fiber direction is increasingly constrained once early post-peak reactions are completed or reduced. In contrast, the transverse FSP is generally higher, with the average level being at 7 compared to 5.6 for the longitudinal, and shows more variation with strong resolvability (t* ~ 4.03 between 121 °C and 204 °C). With the exception of 93 °C, which shows a sudden drop, likely due to the transition in competition between post-cure and onset of deterioration, the DAF values are all above 1, with the highest being at the extremes. The emergence of predominant transverse amplification (i.e., DAF > 1) at this time stage suggests that thermally induced changes in the resin and the fiber matrix interphase influence the frequency dependence of T_g_.

**Figure 12:**
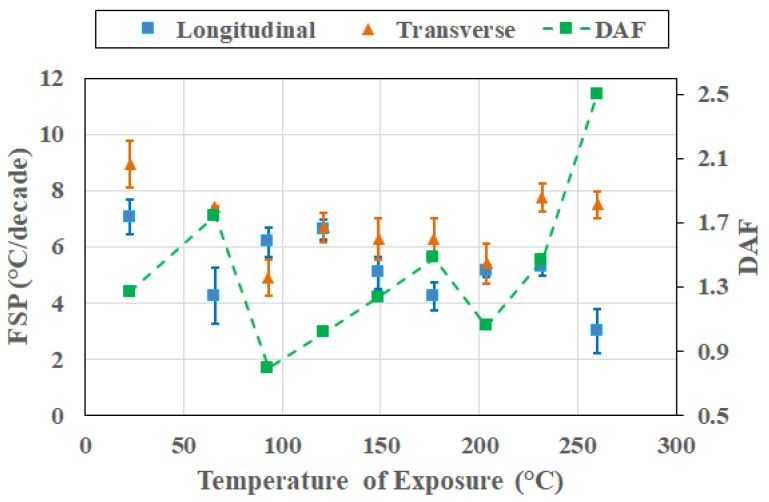
Cross-sectional response after 8 h of thermal exposure as a function of the temperature of exposure.

While directional asymmetry becomes more pronounced, yet temperature selective, at the 4 h level, frequency–direction coupling reaches its most pronounced expression after 8 h of exposure, as seen in Figure 12. Longitudinal FSP is below that of transverse FSP at all temperatures except 93 °C and has relatively low values at the higher temperatures of exposure decreasing from a level of 6.606 at 121 °C to 2.998 at 260 °C, while transverse FSP shows a minor decreasing trend between 121 °C and 204 °C with values of 6.683 and 5.412, respectively, before increasing to levels above 7 at the two highest temperatures. It is noted that at 260 °C, the difference between directions is the largest observed across all time periods, with the highest DAF in the study of 2.5, accompanied by clearly detectable directional separation (t* ~ 4.9). Physically, this regime reflects the combined effects of oxidation-driven resin and interphase degradation at higher temperatures, with the fiber-imposed constraint-based post-cure at lower temperatures, which together maximize directional contrast in relaxation time-scale sensitivity.

**Figure 13:**
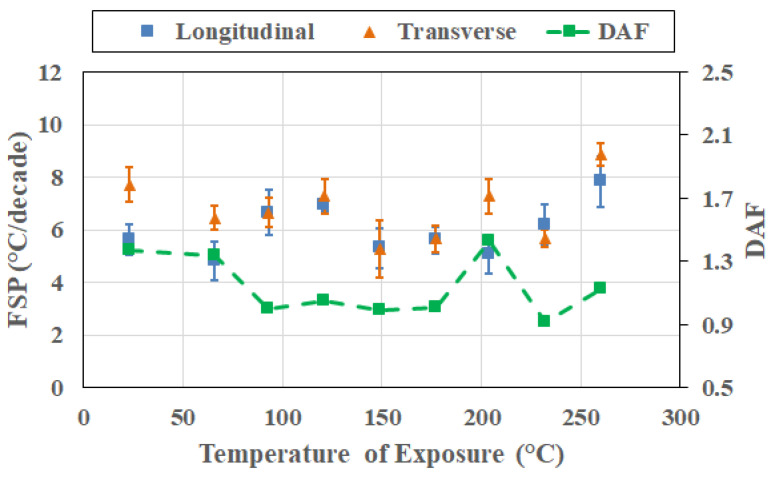
Cross-sectional response after 16 h of thermal exposure as a function of temperature of exposure.

In comparison to the lower time periods of exposure, the 16 h level encompasses three very different regimes, as seen in Figure 13. In the 23 °C to 121 °C range, the responses are representative of increasing levels of post-cure with peak T_g_, and hence maximum post-cure is attained at much longer periods of exposure (potentially greater than 72 h, since the current investigation only extended to 72 h). At 149 °C, peak T_g_ is attained at 16 h and, as discussed earlier, represents the highest levels of T_g_ recorded in both the longitudinal and transverse directions at the levels of 122.17 °C and 120.8 °C, respectively, at 1 Hz. Within the exposure range of 204–260 °C, this is attained at 2 h or less of exposure. Thus, comparisons of metrics across the range of temperatures must be considered as being indicative of more than just temperature effects, and rather as temperature plus “stage of aging” effects, which intrinsically increase variability and reduce transitivity. As seen in Figure 13, longitudinal and transverse FSP values converge within the 93 °C to 177 °C intermediate temperature range with the DAF values near unity. This convergence reflects a balance between post-cure progression, which tends to reduce frequency sensitivity, and incipient degradation, which results in the broadening of the relaxation spectrum. Transverse FSP is higher than longitudinal FSP at the two ends of the temperature spectrum and reaches its highest value of 8.86 at the most severe temperature exposure level of 260 °C. Resolvability analysis shows marginal resolvability for the initial section between 23 °C and 66 °C with clear resolvability (t* ~ 3.09) from the intermediate-to-high, i.e., 149 °C to 260 °C, levels in the transverse direction, with a similar level for the longitudinal direction between a smaller subset range of 204 °C to 260 °C, and with the mid-range where there is convergence reflecting a non-resolvable difference.

**Figure 14:**
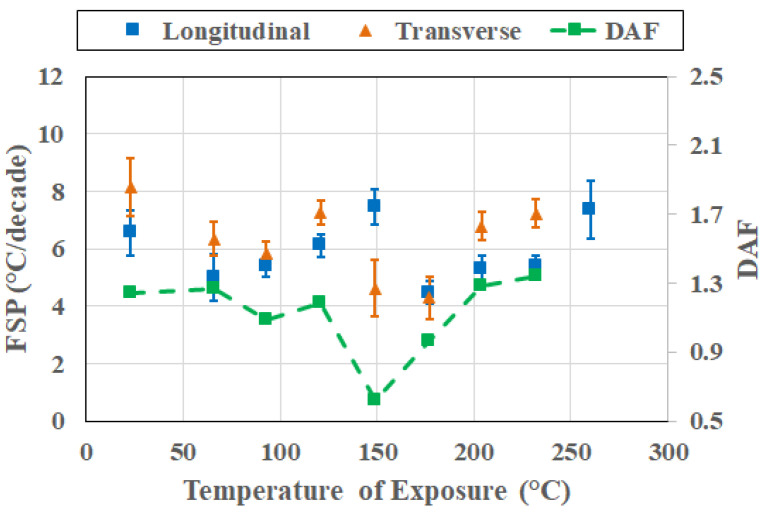
Cross-sectional response after 24 h of thermal exposure as a function of temperature of exposure.

By the 24 h level, frequency sensitivity response becomes more complex, as seen in Figure 14. It should be noted that by this time of temperature exposure the rate of increase in T_g_ at temperatures till 121 °C has decreased with T_g_ levels being close to peak T_g_, while deterioration is noted at temperatures of 149 °C and higher. With the exception of the temperature range between 149 °C and 173 °C, the transverse FSL is higher than longitudinal FSL, with the peak transverse FSL being at 149 °C, which also denotes the lowest value of DAF indicative of the transition between post-cure-based T_g_ increase, and resin and interphase deterioration due to the higher temperature exposure levels. Longitudinal FSP shows a marked increase between 66 °C and 149 °C and between 177 °C and 260 °C, with both ranges showing marginal resolvability at t* levels of 2.412 and 2.613, respectively. In comparison, the transverse FSP is not resolvable (t* = 1.508) in the first range but is clearly resolvable in the second (t* = 3.326) between 177 °C and 232 °C. Physically, the 24 h regime reflects the competition between rising longitudinal sensitivity due to erosion of fiber constraint effectiveness and the evolving transverse sensitivity (i.e., increasing effect of resin and interphase level deterioration). This transverse sensitivity is reflected in significant drops in shear- and other resin-dominated mechanical characteristics in this temperature range [8,19].

**Figure 15:**
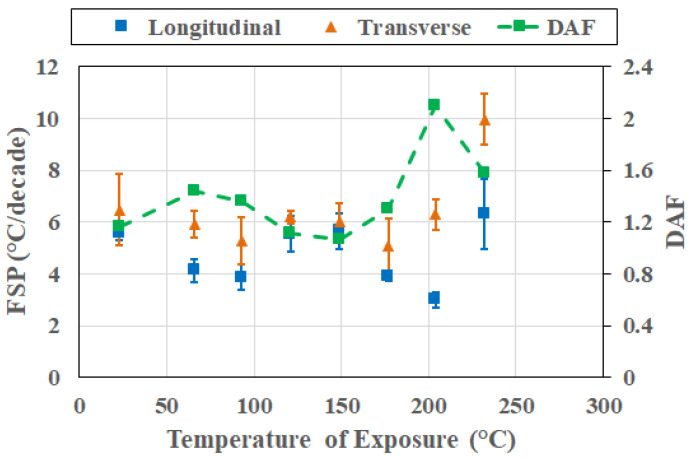
Cross-sectional response after 48 h of thermal exposure as a function of temperature of exposure.

At the 48 h level, thermally induced deteriorated processes clearly dominate the overall response, with directional coupling being strong. As seen in Figure 15, transverse FSP is higher than the longitudinal FSP at all temperatures, resulting in DAF values being greater than unity and reaching levels as high as 2.1 at 204 °C. Differences in longitudinal FSP are clearly differentiable with t* = 3.596 between 149 °C and 204 °C, whereas those for transverse FSP are clearly differentiable with t* = 3.24 between 204 °C and 232 °C. This behavior of directional coupling indicates that degradation-driven heterogeneity and interphase weakening increasingly dominate transverse relaxation. The emergence of strong coupling after the more complex response seen after an exposure period of 24 h emphasizes that frequency–direction coupling can strengthen post-degradation initiation, reinforcing the view that coupling evolves through changes in response regime, especially in light of competition between thermally induced mechanisms at the constituent and interphase levels rather than merely monotonically with period of thermal exposure at elevated temperatures.

**Figure 16:**
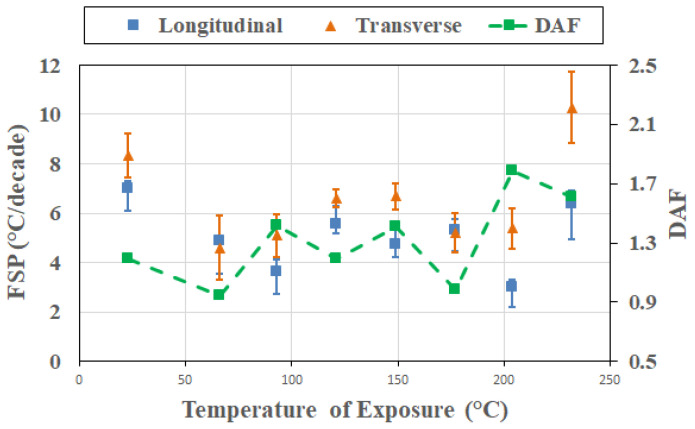
Cross-sectional response after 72 h of thermal exposure as a function of temperature of exposure.

The 72 h level indicates a degradation-dominated regime with strong transverse amplification due to resin-based deterioration. Overall, longitudinal FSP values decrease with an increase in temperature, with some level of variation, and then regain magnitude at the highest level of 232 °C. As seen in Figure 16, transverse FSP values follow the T_g_ trend but are generally higher than the corresponding longitudinal FSP, consistent with advanced degradation, oxidation, and interphase level deterioration leading to rate-sensitive relaxation. It is of interest to note that in the longitudinal direction, marginal-to-clear resolvability is seen between 23 °C and 93 °C, with t* = 2.997, and between 204 °C and 232 °C at t* = 2.417, in contrast to the transverse direction FSP, where resolvability t* = 3.242 is only seen at the highest temperature levels.

From an overall perspective, it can be noted that fixed-time cross sections reveal complex, non-monotonic behavior because the same aging time corresponds to different kinetic states across exposure temperatures and directions. Early times (1–2 h) are dominated by rapid post-cure and kinetic reorganization, producing high but unstable frequency sensitivity and even directional inversion. Intermediate times (4–16 h) mark the emergence of temperature-dependent frequency–direction coupling as matrix-dominated and interphase-controlled processes become increasingly influential. At longer times of exposure (48–72 h), degradation-dominated behavior produces strong transverse amplification and reduced longitudinal sensitivity, reflecting constraint loss and heterogeneous relaxation. These observations demonstrate that frequency sensitivity evolves cumulatively and directionally with thermal exposure, and that meaningful interpretation requires peak-anchored and regime-based analysis rather than reliance only on fixed-time cross sections.

Given the importance of assessing differences across these complex states, it is worth considering resolvability based on cross-sectional analysis across the different time periods of exposure, as shown in Table 3, which reveals the non-monotonic, direction-dependent progression in the detectability of frequency sensitivity evolution. At 1 h, no resolvable contrasts are observed in either loading direction, indicating that early-stage kinetics remain below regression resolution. Clearly resolvable differences first emerge at the 2 h level of exposure in both directions for selected intermediate-to-high temperature intervals. At the 4 h level, resolvability persists only in the transverse direction, reflecting early sensitivity of matrix- and interphase-controlled relaxation to thermal exposure. By 8 h, clearly resolvable contrasts are again observed in both directions, marking the transition from post-cure-dominated to degradation-influenced behavior. At 16 and 24 h levels, resolvability weakens in the longitudinal direction while remaining stronger in the transverse direction, which is consistent with matrix- and interphase-dominated degradation leading to stabilization of fiber constraint kinetics and continued transverse heterogeneity. By the levels of 48 and 72 h, clearly resolvable longitudinal contrasts dominate, reflecting cumulative degradation-driven evolution. Together, these results demonstrate that frequency–directional coupling develops asymmetrically in time and cannot be inferred from single cross sections or monotonic temperature trends.

It is emphasized that Table 3 shows results across each cross-section, and hence, does not look at localized regions, within which, as discussed earlier, better levels of resolvability may exist.

### 3.3. Arrhenius-like Scaling of Frequency Sensitivity

To further assess the temperature dependence of FSP, peak-anchored FSP values were examined as a function of exposure temperature using an Arrhenius-type representation:

(7) $$FSP=Ae^{\frac{{-E}_{app}}{RT}}$$

where T is the absolute temperature in degrees Kelvin, R is the universal gas constant, and E_app_ is a representation of the temperature dependence of frequency sensitivity. When plotted as ln (FSP) versus the inverse of the absolute temperature, both longitudinal and transverse data exhibit near-linear trends over the temperature range where a distinct tan d peak could be identified, resulting in apparent activation energies of 1.8 kJ/mol and 1.3 kJ/mol in the longitudinal and transverse directions, respectively. These activation energies should not be interpreted as molecular relaxation energies normally associated with the use of the Arrhenius equation, but rather as phenomenological descriptors reflecting the temperature dependence. The lower apparent activation energy observed in the transverse direction is consistent with its greater sensitivity to early-stage interphase degradation and oxidation-induced heterogeneity, whereas the longitudinal response remains partially constrained by the fiber architecture.

While the current Arrhenius-type representation provides a useful means for examining the temperature dependence of frequency sensitivity, it should be emphasized that the applicability is inherently limited by the evolving physical state of the material. At low and intermediate exposure temperatures, where relaxation remains relatively homogeneous and tan d peaks are well-defined across frequencies, FSP exhibits approximately Arrhenius-like scaling with temperature. However, at the highest temperature and extended exposure times, deviations from linear Arrhenius behavior are expected as thermo-oxidative degradation, interphase damage, and constraint loss introduce strong spatial heterogeneity. Under these conditions, the assumptions underlying Arrhenius scaling, namely a single dominant activation process and a uniform relaxation environment, are no longer strictly valid. This breakdown is evidenced experimentally by increasing scatter in FSP and divergence between longitudinal and transverse responses. Accordingly, the apparent extracted activation energies should be interpreted as effective kinetic descriptors that are valid within a limited temperature range, rather than intrinsic material constants, and their primary value lies in comparative interpretation between directions and exposure conditions rather than absolute quantification.

### 3.4. Overall Interpretation of Frequency–Directional Coupling and Competing Thermally Induced Phenomena

Given the complexity and range of interactions, and results, based on frequency–directional coupling and the effects of both time and temperature of exposure, it is of interest to develop an overall schematic that could enable the linking of frequency sensitivity, directionality, and thermally induced processes of post-cure progression and deterioration as a means of better comprehending the evolution of T_g_ as a function of time and temperature of exposure. Results discussed in the previous sections enable the overall response to be classified into three zones of early stage, intermediate, and longer/severe exposure.

During the initial stages of exposure, the evolution of glass transition behavior is dominated by post-cure progression and network equilibration. Phenomena of crosslinking, reduction in residual free volume, and redistribution of molecular mobility with the increase in the degree of polymerization lead to increases in the level of T_g_. Fiber-imposed constraints to longitudinal deformation and matrix-dominated relaxation in transverse deformation remain effectively coupled at this stage, producing DAF values near unity and directional differences that are typically below statistical resolvability. In this initial phase, multi-frequency T_g_ measurements primarily reflect the intrinsic kinetic nature of the glass transition rather than exposure-induced homogeneity, with the regime corresponding to post-cure domination with uniform evolution of relaxation kinetics and minimal frequency–directional coupling. With an increase in time and temperature of exposure, the nature of the competition between the positive features of post-cure and the negative aspects of thermally induced resin- and interphase-degradation shift giving rise to an intermediate regime that is characterized by the emergence of direction-dependent frequency sensitivity. Mechanisms of thermally activated oxidation, interphase modification, and bulk resin deterioration introduce heterogeneity and interacting mechanisms of deterioration. These changes are first seen through tests in the transverse direction, where response to excitation is less constrained by fibers and hence shows more sensitivity to changes in molecular mobility and localized deterioration. Consequently, transverse frequency sensitivity often manifests earlier and more strongly than longitudinal frequency sensitivity, leading to elevated directional amplification at selected exposure conditions. Importantly, this amplification is not uniform across temperature or time, but appears in discreet “windows” where competing mechanisms diverge more strongly between directions. The non-monotonic nature of directional amplification in this regime is a direct consequence of competing processes. While heterogeneity tends to increase frequency sensitivity, ongoing post-cure or partial constraint retention can counteract or delay similar changes in the longitudinal response, due to which there exist local areas of convergence of longitudinal and transverse FSP with localized maxima in DAF. This intermediate regime represents a transition between post-cure and degradation-dominated phases, resulting in maximum sensitivity to the direction of testing and non-monotonic frequency–direction coupling.

At long exposure times and especially higher temperature levels, degradation processes increasingly dominate the viscoelastic response. Oxidative chain scission, network degradation, and interphase damage progressively erode the mechanical constraint imposed by fiber architecture, and as a result, longitudinal relaxation becomes more sensitive to excitation frequency. In the transverse direction, results are consistent with advanced degradation in the bulk resin and at the interphase-level, which produces highly heterogeneous relaxation behavior. The coexistence of measurable longitudinal frequency sensitivity and degraded transverse T_g_ highlights the inherent directional nature of thermal exposure in composites and underscores that frequency–direction coupling evolves not just as a result of severe exposure conditions but also through the progressive loss of constraint and increasing relaxation heterogeneity. This state reflects a degradation-dominated regime in which frequency–direction coupling weakens on inversion, and conventional T_g_ metrics determined through resin-dominated tests can lose integrity.

Temperature and time-regime-based interpretation provides clarification to the obscuring of critical aspects of thermally induced changes in polymer composites when characterized through single-frequency measurement of T_g_. Since T_g_ increases systematically with excitation frequency, a single-frequency measurement samples only a narrow portion of a broader relaxation spectrum. Changes in frequency sensitivity, especially in light of differences between longitudinal and transverse defamation, may thus remain undetected. Multi-frequency DMTA, combined with direction-sensitive analysis, provides a much better interpretation of the nuances of the evolving spectrum and enables detection of subtle, but mechanistically meaningful, changes in network kinetics, interphase conditions, and constraint effects. At the same time, the results delineate the practical limits of T_g_-based metrics, especially in cases where longer and more severe thermal exposures result in significant deterioration in matrix response. It should be emphasized that single-frequency-based assessment of T_g_ effectively collapses distinct relaxation states into a single descriptor that cannot, as such, convey the intricate complexities of directional differences.

**Figure 17:**
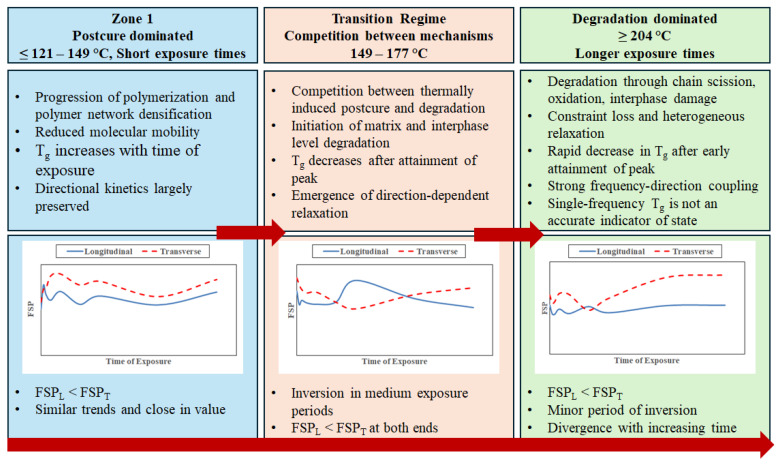
Regime-based framework for overall (T, t) response.

The overall response can be described schematically within a regime-based framework, as in Figure 17, wherein the early regime characterizes a response governed by post-cure-dominated kinetics, weak directional dependence, and clarity in frequency sensitivity. The intermediate, or transitional, regime is characterized by the emergence of relaxation heterogeneity, enhanced transfer sensitivity due to increasing resin, and interphase deterioration, some of which is offset by increasing effects of post-cure, and localized directional amplification. The final regime is dominated by degradation-driven constraint loss, non-monotonic directional behavior, and eventual loss of transverse T_g_ integrity. The schematic shown in Figure 17 consolidates these, emphasizing the complex interplay and evolution of T_g_.

## 4. Summary and Conclusions

This study presents a systematic investigation of the evolution of glass transition temperature and its frequency–direction coupling in a wet-layup unidirectional carbon/epoxy composite representative of systems used in rehabilitation and strengthening, as well as in components for civil, offshore, and naval infrastructure, subjected to thermal exposure conditions across a wide range of temperatures and times. Multi-frequency DMTA measurements from both longitudinal and transverse flexural configurations are used in conjunction with metrics of frequency sensitivity, FSP, and directional amplification, DAF, to interrogate and advance the physical interpretation of thermally induced polymer network evolution in these reinforced composite systems. The response is further partitioned into early- and late-stage aging regimes to differentiate post-cure-dominated behavior from degradation-dominated response. By explicitly treating T_g_ as a function of temperature, time of exposure, frequency, and fiber alignment, this work demonstrates why single-frequency T_g_ measurements are insufficient for aging assessment and provides a mechanistically interpretable representation (coupling map) that links directional viscoelastic evolution to the competing influences of oxidation, interphase evolution, and constraint loss.

### 4.1. Key Findings and Trends

*1. Frequency sensitivity of T_g_ is inherently direction-dependent and evolves non-monotonically with aging time and temperature.* Across all exposure conditions, T_g_ measured from the tan d peak increases monotonically with excitation frequency, which is consistent with the kinetic nature of the glass transition. However, the magnitude of this frequency dependence as represented by the frequency sensitivity parameter, FSP, differs between longitudinal and transverse loading directions and evolves in a manner that cannot be captured by single-frequency T_g_ measurements.

*2. Detectable frequency–directional coupling emerges progressively and asymmetrically in time.* A resolvability framework based on the standard error of the FSP slope reveals that frequency sensitivity differences are not immediately detectable at early aging times. Clearly resolvable contrasts first appear at short times (≈2 h) for selected intermediate–high temperature intervals, persisting preferentially in the transverse direction at intermediate times of 4–16 h, and becoming dominant in the longitudinal direction at longer aging times of 48–72 h. This staggered emergence reflects fundamentally different controlling mechanisms in the two directions.

*3. Cross-sectional evolution highlights path dependence that is obscured in monotonic temperature or time trends*. Cross sections taken at fixed times of exposure demonstrate that large temperature spans do not guarantee resolvable differences in frequency sensitivity, even at long exposure times. The absence of transitivity across temperature intervals underscores that the evolution of FSP is governed by the aging and deterioration state of the polymer network rather than by temperature or time alone.

*4. Peak-anchored analysis provides a rigorous baseline for interpreting subsequent evolution.* Evaluating FSP and DAF at the exposure conditions corresponding to the peak T_g_ attained for each temperature of exposure does not reveal simple monotonic trends with exposure temperature. Instead, peak-anchoring serves as a physically meaningful reference state that isolates the culmination of post-cure processes. Departures from this baseline at later times and higher temperatures provide a clear framework for interpreting degradation-driven evolution without over-interpreting absolute trends of the peak itself.

*5. Transverse sensitivity reflects early evolution of matrix and interphase degradation, whereas longitudinal sensitivity reflects cumulative constraint loss.* The earlier and more persistent resolvability of FSP in the transverse direction is consistent with the dominance of matrix- and interphase-controlled relaxation processes, which are highly sensitive to oxidation, free-volume generation, and heterogeneity. In contrast, the delayed, but ultimately stronger, resolvability in the longitudinal direction reflects the gradual loss of fiber-imposed constraint and the accumulation of deterioration in the bulk matrix and at the interphase level.

### 4.2. Methodological Contributions and Broader Significance

Beyond the specific material system studied, this work demonstrates the necessity of multi-frequency, multidirectional DMTA for the meaningful interpretation of glass transition behavior in composites exposed to extended periods of elevated temperature. The results show explicitly why single-frequency T_g_ measurement, even when tracked as a function of exposure temperature and time, can fail to detect important changes in polymer network dynamics and damage state. The introduction of frequency sensitivity parameters derived from regression across frequencies, standard-error-based resolvability criteria, and cross-sectional and peak-anchored critical frameworks provides a robust and transferable methodology for assessing environmental degradation in polymer composites. These approaches enable statistically defensible discrimination between true material evolution and experimental scatter, addressing a persistent challenge in degradation studies, especially when limited samples may exist due to the large number of exposure conditions to be evaluated.

### 4.3. Implications for Durability Assessment and Design

The findings have direct implications for the durability assessment of composite structures exposed to elevated temperatures, especially as related to materials processed under an ambient/moderate temperature cure, in which polymerization could continue to progress under conditions of low–moderate temperature exposure over the intended service-life. The strong directional dependence and path dependence of frequency sensitivity demonstrate that T_g_ alone is an incomplete descriptor of thermally induced changes in polymer networks. Instead, the evolution of frequency–direction coupling offers a more sensitive indicator of early-stage degradation, interphase evolution, and constraint loss, which precede gross reductions in mechanical performance. In this context, multi-frequency DMTA should be considered not just as a characterization tool, but as a critical part of the diagnostic framework focused on interpreting the underlying physics of aging and degradation in fiber-reinforced polymer composites. The concepts and metrics developed here provide a foundation for future work linking viscoelastic evolution to mechanical property degradation, lifetime prediction, and performance-based design under long-term thermal exposure environments.

A promising future direction could be the development of a time-frequency analog to conventional superposition concepts used in viscoelastic durability modeling. In classical time–temperature superposition, frequency shifts approximate changes in observation time scale for thermo-rheologically simple materials. Here, because T_g_ increases systematically with excitation frequency at each aging condition, each (T, t) state can be viewed as defining a local T_g_(f) relationship with a slope quantified by FSP. In principle, combining these T_g_(f) relationships with the temperature- and time-dependence of FSP could enable regime-specific forecasting, such as (i) estimating T_g_ at a reference frequency for untested temperature–time conditions, and (ii) predicting the time of service required for T_g_ to reach a specified threshold at a given exposure temperature, which could be of critical importance in determining service life for composite materials exposed to thermal environments below peak T_g_ for that material during normal use over their service-life. However, unlike classical superposition, any shift factors would be state-dependent and direction-specific, and breakdowns are expected in regimes where degradation induces strong heterogeneity or where tan d integrity is compromised due to severe degradation. Consequently, temperature-frequency predictive approaches may need to be implemented as piecewise/regime-change-limited models validated within the domain where T_g_ peaks remain well-defined.

## Figures and Tables

**Table 1:** Time of exposure pertaining to attainment of peak T_g_ as a function of temperature and frequency of excitation.

Temperature of Exposure (°C)	Longitudinal Direction						Transverse Direction					
	Time (Hours)	0.3 Hz	1 Hz	3 Hz	10 Hz	30 Hz	Time (Hours)	0.3 Hz	1 Hz	3 Hz	10 Hz	30 Hz
66	72	97.44	100.12	102.62	104.43	107.45	72	103.80	104.01	104.15	108.76	112.96
93	72	114.25	114.41	116.25	118.76	121.10	72	114.45	116.40	118.20	119.91	125.47
121	48	115.50	120.24	120.44	124.14	127.38						
	72	115.58	120.85	120.80	124.97	127.33	72	115.42	119.59	122.73	126.48	128.46
149	16	115.91	121.17	121.27	124.39	127.53	16	118.98	120.80	120.93	125.23	129.92
177	8	111.83	112.74	114.90	117.05	120.28	8	110.29	112.77	114.83	118.10	123.30
	16	111.00	112.60	114.97	118.88	121.93	16	111.23	114.02	115.42	120.08	122.32
							24	111.97	114.42	114.56	117.34	121.24
204	1	107.97	113.08	115.17	116.09	119.15	1	107.31	109.34	113.21	115.93	119.74
	2	112.34	112.70	114.14	117.68	120.09	2	110.84	112.43	113.14	115.92	119.02
232	1	107.48	112.91	113.27	115.39	119.29	1	109.71	111.39	114.93	119.31	122.27
260	1	101.15	103.40	104.45	109.00	112.31	1	101.22	103.42	104.86	108.84	113.96

**Table 2:** Ranges for resolvability, t*.

t* Range	Interpretation	Description
t* < 2	Not resolvable	Differences are comparable to the level of uncertainty
2 ≤ t* < 3	Marginally resolvable	Suggestive of differences but with weak separation
t* ≥ 3	Clearly resolvable	Strong, uncertainty-exceeding difference

**Table 3:** Full Resolvability Matrix (Following Table 2 with Resolvable = at least one range with t* ≥ 3, Marginal = at least one range with 2 ≤ t* < 3, Not resolvable = all t* < 2).

Time of Exposure (Hours)	Longitudinal FSP	Transverse FSP	Interpretation
1	Not resolvable	Not resolvable	Early kinetics are below resolution
2	Clearly resolvable	Clearly resolvable	First detectable divergence
4	Not resolvable	Clearly resolvable	Early transverse sensitivity
8	Clearly resolvable	Clearly resolvable	Transition to coupled evolution
16	Marginally resolvable	Clearly resolvable	Directional asymmetry peaks
24	Not resolvable	Marginally resolvable	Longitudinal stabilization
48	Clearly resolvable	Clearly resolvable	Late-stage degradation dominance
72	Clearly resolvable	-	Longitudinal accumulation only

## Data Availability

The original contributions presented in this study are included in the article. Further inquiries can be directed to the corresponding author.
